# Expanded Latissimus Dorsi Myocutaneous Flap for Burn Scar Reconstruction

**Published:** 2018-07-13

**Authors:** Angie Zhang, Amra Kuc, Lillian Tung, Kathryn S. King, Deniz Dayicioglu

**Affiliations:** ^a^University of South Florida Morsani College of Medicine, Tampa; ^b^Department of Plastic Surgery, University of South Florida Morsani College of Medicine, Tampa

**Keywords:** pre-expanded latissimus dorsi myocutaneous flap, burns, burn scars reconstruction, neck scar contracture, upper extremity burn scar reconstruction

## DESCRIPTION

A 41-year-old woman with a history of flame burns to her neck, chest, abdomen, and upper and lower extremities bilaterally presented to the clinic for reconstruction following bilateral mastectomy for breast cancer. She subsequently underwent excision of contractures on her neck and left upper extremity with reconstruction using bilateral pre-expanded latissimus dorsi (LD) myocutaneous flaps.

## QUESTIONS

1. What is the pathophysiology behind the formation of burn scar contractures?
2. What are the options for reconstruction of neck contractures?
3. What are the options for reconstruction of upper extremity contractures?
4. What is the pre-expanded LD concept?

## DISCUSSION

Burn injuries remain a common cause of traumatic injuries worldwide and can lead to significant deformity secondary to scar contractures. After full-thickness skin loss, wound contraction and epithelialization from the margins lead to contractures.^1^ Risk factors for developing contractures include a prolonged inflammatory state, high microbial burden, increased burn depth, wound or incision site tension, and genetics. A variety of treatments exist, including surgery and CO_2_ laser therapy, with the basic tenet of treatment being tension release. Burn scar contractures of aesthetically and functionally sensitive areas demonstrate a particular challenge to the reconstructive surgeon.

Contractures of the neck may distort facial features and limit neck mobility; therefore, reconstructive goals focus on restoration of neck mobility and function while maintaining pleasing facial aesthetics. Scar contractures may be released and excised, with the resultant defect being reconstructed with skin grafting.^2^^,^^3^ Pedicled flaps from the chest, back, or cervicohumeral region may be also used for reconstruction and demonstrate a lower risk of postoperative contracture as compared with skin grafting. Pre-expansion of both locoregional and free flaps offers several advantages including increasing flap size and thinning of tissue for better contour, which holds particular importance in neck reconstruction.^4^

Similarly, upper extremity contractures have significant morbidity to function and mobility. Reconstructive options for upper extremity contractures include skin grafts, local skin/fasciocutaneous/muscle flaps, and free flaps.^5^ Full-thickness skin grafts are preferred over split-thickness grafts, as the former gives a more durable and cosmetically pleasing result with a lower risk for recontracture. In the case of an extensive burn, a full-thickness skin graft is impractical and a flap reconstruction is often necessary.^2^ Both local and free flaps have been successfully used in burn contracture reconstruction. The major advantage of using flaps is the low reoccurrence rate.^5^

Tissue expansion is a well-accepted and reliable method to provide extracutaneous tissue in reconstruction. Pre-expansion has also been shown to improve flap vascularity by increasing both the number and caliber of vessels in the flap.^6^ These characteristics of expanded tissue prove worthwhile in burn reconstruction when nonburned/noncontracted tissue can be difficult to find. We describe a patient who underwent reconstruction of burn scar contractures to the neck and left upper extremity using pre-expanded LD flaps. Tissue expanders may be placed under the latissimus muscles and expanded to the desired size.^7^^,^^8^ After expansion and allowing sufficient time for tissue remodeling (maximized at 6-12 weeks following expansion), the LD flap is raised in the standard fashion. The flap can be tunneled through the axilla for use in neck, chest, or upper extremity reconstruction, or it may be used as a free flap for distant reconstruction.

**Figure 1:**
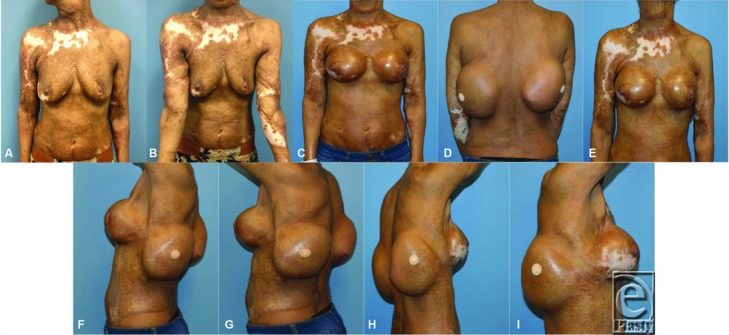
Preoperative photographs demonstrating burn scars to the neck, bilateral upper extremities, chest, and abdomen, with some contraction bands to the neck. Before mastectomy. (a) Anterior view. (b) View of upper extremities. Tissue expanders in place below bilateral latissimus dorsi muscles. (c) Anterior view. (d) Posterior view. (e) Anterior view with neck extended. (f) Left lateral view. (g) Left lateral oblique view. (h) Right lateral oblique view. (i) Right lateral view.

**Figure 2:**
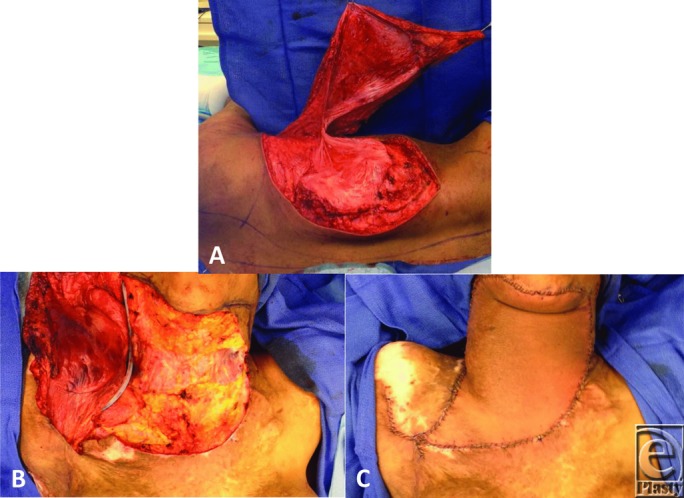
(a) Elevation of the right latissimus dorsi muscle flap. (b) Excision of the burn scar to the anterior neck. Latissimus dorsi muscle flap tunneled through the right axilla for coverage of the anterior neck defect. (c) Inset of the right latissimus dorsi muscle flap to the anterior neck defect.

**Figure 3:**
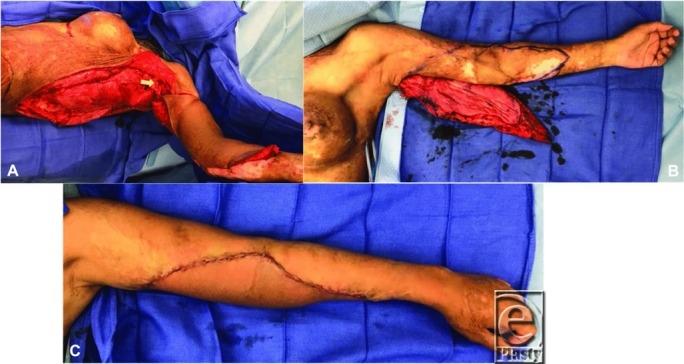
Intraoperative photographs of the left latissimus dorsi flap for reconstruction of the left upper extremity burn scar. (a) Left latissimus dorsi flap elevation. The arrow indicates location of the pedicle. Flap stapled in place over the location of planned burn scar excision. (b) Left latissimus dorsi flap and planned excision of the left upper extremity burn scar. (c) Flap inset.

**Figure 4:**
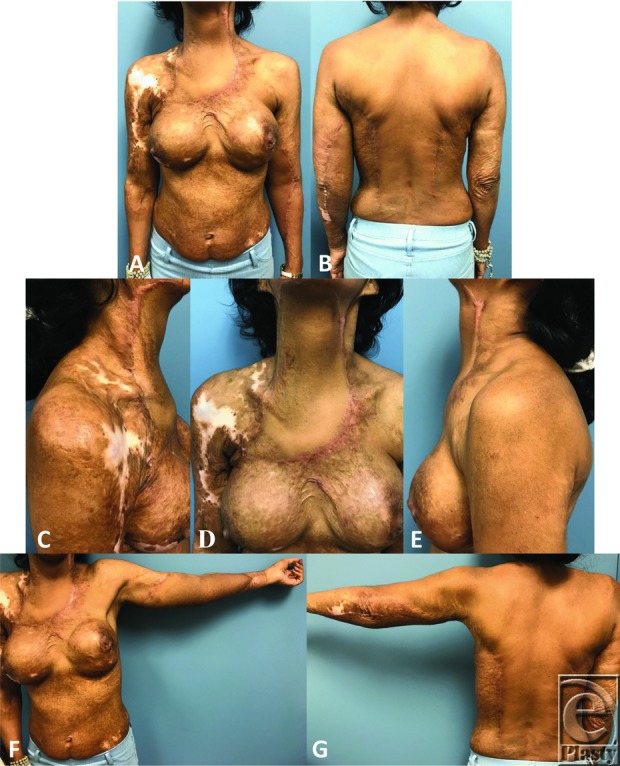
Postoperative photographs following latissimus dorsi flap reconstruction for neck burn scar contracture (5 months postoperative) and left upper extremity burn scar (2 months postoperative). (a) Anterior view. (b) Posterior view. (c) Right lateral view with neck extended. (d) Anterior view with neck extended. (e) Left lateral view with neck extended. (f) Anterior view with upper left extremity extended. (g) Posterior view with upper left extremity extended.

A 41-year-old woman presented to the clinic for discussion of breast reconstruction following a diagnosis of right-sided breast cancer. She reported a history of 80% to 90% total body surface area burns sustained in adolescence. On examination, she was noted to have burn scar contractures to her neck and upper extremities (Figs 1*a*–1*b*). Given the severity of her neck contractures, we decided to place bilateral LD tissue expanders at the time of her mastectomy for later scar revision, in addition to placing breast tissue expanders for delayed breast reconstruction. Postmastectomy, each LD tissue expander was inflated to 1000 cm^3^ to maximize flap dimensions (Figs 1*c*–1*i*). The patient was then taken to the operating room for excision and reconstruction of her neck contractures. The pre-expanded right LD flap was used to cover the neck defect on the left after scar release (Figs 2*a*–2*c*). As we were able to obtain satisfactory neck closure using the right LD flap alone, the patient underwent a second operation to excise and reconstruct her left upper extremity contractures, using the pre-expanded left LD flap (Figs 3*a*–3*c*). Postoperatively, the patient reports doing well with improvement in her mobility and function (Figs 4*a*–4*g*). This case demonstrates the utility of pre-expanded flaps for use in burn scar reconstruction.
